# An analytical model of dynamic sliding friction during impact

**DOI:** 10.1038/srep40102

**Published:** 2017-01-05

**Authors:** Kazuo Arakawa

**Affiliations:** 1 Research Institute for Applied Mechanics, Kyushu University, 6-1 Kasuga, Fukuoka 816-8580, Japan

## Abstract

Dynamic sliding friction was studied based on the angular velocity of a golf ball during an oblique impact. This study used the analytical model proposed for the dynamic sliding friction on lubricated and non-lubricated inclines. The contact area *A* and sliding velocity *u* of the ball during impact were used to describe the dynamic friction force *F*_*d*_ = *λAu*, where *λ* is a parameter related to the wear of the contact area. A comparison with experimental results revealed that the model agreed well with the observed changes in the angular velocity during impact, and *λAu* is qualitatively equivalent to the empirical relationship, *μN* + *μη*′*dA/dt*, given by the product between the frictional coefficient *μ* and the contact force *N*, and the additional term related to factor *η*′ for the surface condition and the time derivative of *A*.

Golf, one of the most popular sports worldwide, has a long history and the physics of golf has been studied for centuries^1^,^2^. Aerodynamic studies of golf balls have demonstrated the following fundamental mechanisms. A dimpled golf ball flies much farther than a smooth ball due to turbulence caused by the dimples. The spin of the ball, imparted by the club, changes the direction and flying distance of the ball in the air. The impact dynamics of golf balls^3^ have also been studied (Fig. 1a). For example, previous studies investigated the oblique impact of the ball with a steel target^4^ (Fig. 1b) and revealed that the sliding (*u*) and angular (*ω*) velocities during impact increased with the inbound velocity *V*_*i*_. The coefficient of restitution and contact time on the target decreased gradually with *V*_*i*_. An impact study using a transparent polymethyl methacrylate (PMMA) target^5^ with dry and oiled surfaces (Fig. 1c) showed that lubrication with oil did not affect the contact area or time. The sliding velocity *u* increased, while the angular velocity *ω* decreased.

A study of the angular velocity *ω* demonstrated the following^6^: (i) the experimental value of *ω* increased in the initial phases of contact and then decreased; (ii) there was a significant discrepancy between the experimental results and analytical velocity *ω* derived from *μN*; and (iii) the experimental results agreed with the analytical velocity *ω* given by *μN* + *μη*′*dA/dt*. Many studies have investigated the dynamic contact problem from analytical and experimental perspectives^7^,^8^,^9^,^10^,^11^ to elucidate influential factors, including the contact force^12^,^13^,^14^,^15^,^16^,^17^, contact area^18^,^19^,^20^,^21^, sliding velocity^14^,^15^,^17^,^22^,^23^,^24^,^25^, surface roughness^18^, temperature^26^,^27^, humidity^22^,^24^,^28^, and interface wear^13^,^29^. However, these effects are still not fully understood.

To study influential factors, sliding tests conducted under different surface conditions demonstrated the following: (i) the sliding velocity of polyurethane (PU) rubber on oiled inclines^30^ was significantly dependent on the contact area; (ii) the contact area of polytetrafluoroethylene (PTFE) spheres on dry inclines^31^ increased with wear, while the sliding velocity decreased; and (iii) the analytical model indicated that the contact area and sliding velocity are key factors on oiled surfaces^30^, while the wear of the contact surface can also be an influential factor on dry surfaces^31^, implying that the dynamic friction force can be expressed as *F*_*d*_ = *λAu*.

This work studied dynamic sliding friction based on the angular velocity *ω* of a golf ball during an oblique impact. The applicability of the frictional force *F*_*d*_ = *λAu* proposed for the dynamic sliding tests^30^,^31^ to the impact problem was examined, together with the reported correlation with the empirical relationship^6^ *μN* + *μη*′*dA/dt*.

Figure 2a shows the contact area *A* of the ball as a function of time *t*. For the inbound ball velocity *V*_*i*_ = 32 m s^−1^, the value of *A* increased in the early phases of impact, attained a peak value of 150 mm^2^ at *t* = 220 *μ*s, and then decreased subsequently. To simplify the analysis, the contact area *A* was expressed as follows:


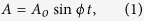


where *A*_*o*_ is the peak value of *A, ϕ* = π/*t*_*c*_, and *t*_*c*_ is the contact time. The results in Fig. 2a show that equation (1) agrees with the experimental data. A similar equation was previously used for erosive wear problems to describe the penetration depth of abrasive spherical particles into solid surfaces^32^.

The rotation angles *θ* for *V*_*i*_ = 28 and 61 m s^−1^ are shown in Fig. 2b, where the experimental results are plotted using symbols; the curves derived from a data-fitting procedure are also shown. For two inbound velocities, *θ* increased slightly in the initial phases of contact, significantly in the subsequent phases, and then gradually in the final phases. The largest values of *θ* for *V*_*i*_ = 28 and 61 m s^−1^ were 13 and 26°, respectively. The angular velocity *ω*_*e*_ was obtained by differentiating the fitted curve *θ(t*) with respect to time, *t* (Methods).

The angular velocities *ω*_*e*_ for *V*_*i*_ = 28 and 61 m s^−1^ are shown in Fig. 3a and b, respectively. For both inbound velocities, *ω*_*e*_ increased in the initial phases of contact and then decreased in the subsequent phases. The peak value of *ω*_*e*_ for *V*_*i*_ = 28 m s^−1^ was 6,700 rpm at *t* = 260 *μ*s, and it decreased significantly to 4,300 rpm just before rebounding at *t*_*c*_ = 500 *μ*s. As *V*_*i*_ increased to 61 m s^−1^, the peak value of *ω*_*e*_ increased to 15,400 rpm at *t* = 240 *μ*s, and then decreased markedly to 9,800 rpm just before rebounding at *t*_*c*_ = 460 *μ*s.

To study the rotation behaviour, the analysis used the following assumptions: (i) the target is rigid and the ball with mass *m* (46 g) and radius *r* (21.3 mm) is treated as a hard sphere; (ii) the contact area *A* is represented with equation (1); and (iii) the gravity force and rolling friction acting on the ball can be disregarded. The angular velocity ω* is given as follows:


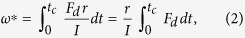


where *I* (=2 m*r*^2^/5) is the inertia moment of the ball about the centre of rotation.

For lubricated friction^30^, we expressed the dynamic friction force, *F*_*d*_* *=* τA*, using the Couette flow shear stress *τ* = (*η*/*h)u*, where *η* and *h* are the viscosity and thickness of the oil layer on the contact area, respectively. Therefore, the dynamic friction force *F*_*d*_ can be given as follows:


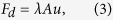


where *λ* = *η*/*h* and the units of *λ* are Pa∙s∙m^−1^.

The oblique impact of a golf ball caused wear of the ball surface in the contact area; hence, this analysis used equation (3), assuming that λ is related to the wear property of the contact area^30^ during impact. The sliding motion of the ball is expressed as follows:


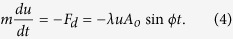


Using the separation of variables and integrating equation (4), assuming that *λ* is constant, we obtain:


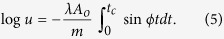


Integrating equation (5) with respect to *t* for *u *=* u*_*o*_ at *t* = 0, the sliding velocity *u* can be expressed as follows:


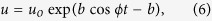


where *b* (=*λA*_*o*_/*m ϕ*) is a dimensionless parameter. Under the assumption of a relatively large mass *m* and a very short duration *t*_*c*_, we approximated exp(*b* cos *ϕ* *t* − *b*) in equation (6) as (1 + *b *cos *ϕt *− *b*) using a series expansion, and represented the sliding velocity *u* as follows:


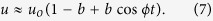


Substituting equations (1) and (7) into equation (3), equation (2) can be rewritten as follows:


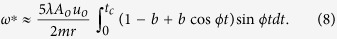


Integrating equation (8) with respect to *t* for ω^*^ = 0 at *t* = 0, the angular velocity ω^*^can be expressed as follows:


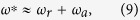


where


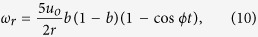



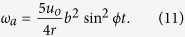


Equation (10) corresponds to the solution given by the hard-sphere model and equation (11) relates to the effect of the change in contact area^6^.

The analytical results of equation (9) for *V*_*i*_ = 28 and 61 m s^−1^ are shown in Fig. 3a and b, respectively. The assumed values of the coefficients for *V*_*i*_ = 28 m s^−1^ were *u*_*o*_ = 12 m s^−1^, *t*_*c*_ = 500 *μ*s, *A*_*o*_ = 130 mm^2^, and *λ* = 1.81 × 10^6^ Pa∙s∙m^−1^ (therefore, *b* = 0.81). For *V*_*i*_ = 61 m s^−1^ they were *u*_*o*_ = 27 m s^−1^, *t*_*c*_ = 460 *μ*s, *A*_*o*_ = 270 mm^2^, and *λ* = 0.96 × 10^6^ Pa∙s∙m^−1^ (therefore *b* = 0.83). Equation (9) agreed well with the experimental result for *V*_*i*_ = 28 m s^−1^. However, the model yielded slight discrepancies from the experimental result for *V*_*i*_ = 61 m s^−1^, probably due to the influence of ball deformation during impact. Two things should be noted: (i) the values of *u*_*o*_ were slightly smaller than those determined theoretically with *u*_*o*_* *=* V*_*i*_ sin*θ*_*i*_, which can be attributed to the energy loss due to the internal friction of the ball at impact^4^, and (ii) similar values of *b* were obtained for both inbound velocities, suggesting that there is an inverse relationship between *A*_*o*_ and *λ*.

To study the effect of *λ* on *A* and *u*, we made a cursory examination of the values of ω^*^ at *t *=* t*_*c*_/2 based on the following approximations: (i) from equation (9) ω^*^ ~ 5*u*_*o*_*b*(2 − *b)/*4*r*; (ii) for different state *ω*_s_^*^ ~ 5*u*_*s*_*b*_*s*_(2 − *b*_*s*_)*/*4*r*, where *b*_*s*_ = *λ*_*s*_*A*_*s*_/*mϕ*_*s*_; and (iii) assuming that *ϕ*_*s*_ ~ *ϕ* and *b*_*s*_ ~ *b, ω*_s_^*^/ω^*^ ~ *u*_*s*_*b*_*s*_*/u*_*o*_*b* was obtained. The effect of *λ* was examined based on *ω*_s_^*^/ω^*^ = 1. For *A*_*s*_* *>* A*_*o*_ and *u*_*s*_* *=* u*_*o*_, *ω*_s_^*^/ω^*^ ~ *λ*_*s*_*A*_*s*_/*λA*_*o*_, resulting in *λ*_*s*_* *<* λ*; while for *A*_*s*_* *=* A*_*o*_ and *u*_*s*_* *<* u*_*o*_, *ω*_s_^*^/ω^*^ ~ *λ*_*s*_*u*_*s*_*/λu*_*o*_, resulting in *λ*_*s*_* *>* λ.* Similar relations can also be determined using equation (3), i.e., *λA*_*o*_*u*_*o*_ = *λ*_*s*_*A*_*s*_*u*_*s*_. This suggests that *λ, A,* and *u* are correlated with each other during dynamic sliding.

The dynamic sliding friction was related to *μN* + *μη*′*dA/dt* in a previous study^6^. To clarify the correlation with the present model, a cursory examination was made of equation (3) using the following approximations: (i) *u* ~ *u*_*o*_ (1 − *b* + *b* cos *ϕt*) in equation (7); (ii) differentiating equation (1) with respect to *t*, we obtain  cos *ϕt* = (*ϕ*/*A*_*o*_)*dA/dt*; and (iii) the relation *F*_*d*_ ~ *λu*_*o*_ (1 − *b)A* + *λu*_*o*_ *bϕ (A*/*A*_*o*_)*dA/dt* was obtained. This approximate expression for equation (3) is qualitatively equivalent to the previous relationship, since *A* depends on *N*. This implies that equation (3) more closely describes the dynamic friction force during an oblique impact.

## Methods

A golf ball was launched horizontally with an air gun so that it obliquely struck a target rigidly clamped and vertically inclined at an angle of 30°. This study used three-piece golf balls, with a mass of 46 g and diameter of 42.6 mm. A high-speed video camera (HPV-1; Shimadzu) was used to record 100 frames as bitmap graphics (size 312 × 260 pixels) at a framing interval of 10 *μ*s. The impact tests were performed at room temperature for a ball inbound at a velocity between 28 and 61 m s^−1^. The target surface was degreased with alcohol, and new balls were used to minimise the change in roughness of the ball surface. On oblique impact with the transparent PMMA target (size 130 × 170 mm^2^, thickness 20 mm), the contact area became dark due to diffuse reflection, and the concave surfaces of the dimples on the ball surface barely contacted the target. The contact area was determined by subtracting the noncontact area due to the dimples using image analysis. The rotation angle of the ball on the steel target (diameter 40 mm, thickness 10 mm) was determined as follows: (i) an image of the ball before impact was selected; (ii) two vertical lines were drawn from the ball centre parallel to the target; (iii) the four markers closest to these two lines and farthest from the ball centre were selected, and the rotation angles of the four markers were measured as a function of time; and (iv) we assumed that the average value of the four angles was the rotation angle of the ball. To minimise data scattering in the evaluation of the angular velocity, we used a data-fitting procedure based on the least-squares method; the measured values of ball rotation were expressed as a ninth-order polynomial of time to fit the observed values. The angular velocity was determined from the first time derivative of the fitted curve.

## Additional Information

**How to cite this article:** Arakawa, K. An analytical model of dynamic sliding friction during impact. *Sci. Rep.* **7**, 40102; doi: 10.1038/srep40102 (2017).

**Publisher's note:** Springer Nature remains neutral with regard to jurisdictional claims in published maps and institutional affiliations.

## Figures and Tables

**Figure 1:**
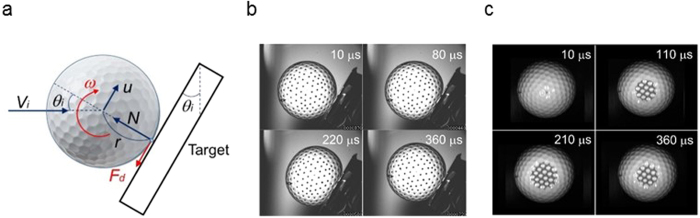
Impact behaviour of a golf ball. (**a**) The rotating and sliding motion of the ball during impact. (**b**) High-speed images of the ball hitting a steel target at an impact velocity *V*_*i*_ = 28 m s^−1^. Markings were made on the dimples to enable ball surface measurement. (**c**) High-speed images of the ball hitting a transparent polymethylmethacrylate (PMMA) target at *V*_*i*_ = 32 m s^−1^. The images were photographed from the reverse side of the target.

**Figure 2:**
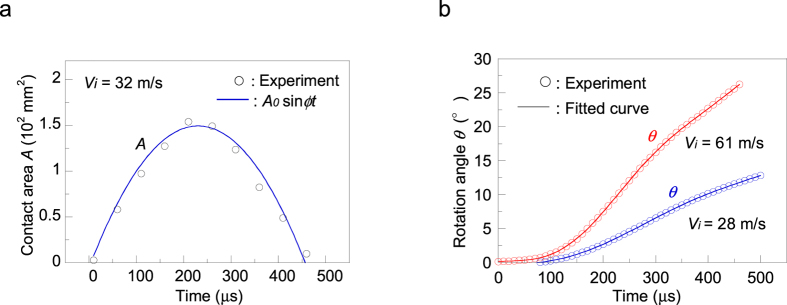
Contact and rotation behaviour of the ball. (**a**) Contact area *A* versus time *t*; the experimental results are plotted with symbols. The contact area *A* was expressed as *A*_*o*_ sin *ϕ* *t*, where *A*_*o*_ is the peak value of *A, ϕ* = π/*t*_*c*_, and *t*_*c*_ is the contact time. (**b**) Rotation angle *θ* versus time *t*. For the two inbound ball velocities, *θ* grew slightly in the initial phases of contact, significantly in the subsequent phases, and then gradually in the final phases.

**Figure 3:**
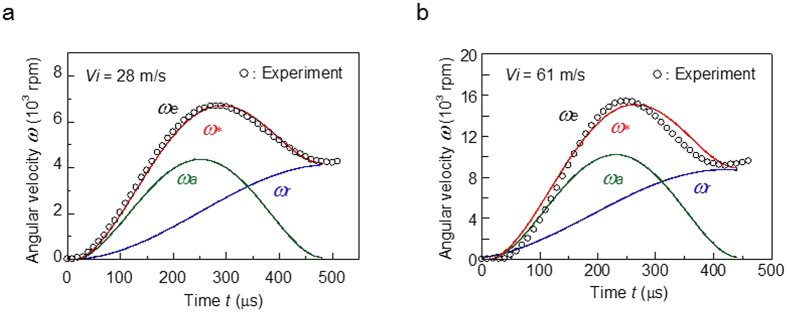
Angular velocities of the ball during impact. (**a**) Angular velocity *ω*_*e*_versus time *t* during impact for *V*_*i*_ = 28 m s^−1^. *ω*_*e*_ increased in the initial phases of contact and then decreased. The peak value of *ω*_*e*_was 6,700 rpm, and this gradually decreased to 4,300 rpm just before rebounding. The angular velocity ω^*^(=*ω*_*r*_* *+* ω*_*a*_) derived from the proposed model was comparable with the experimental results, where *ω*_*r*_and *ω*_*a*_were related to the angular velocities given by a rigid-sphere model and the change in the contact area, respectively. (**b**) Angular velocity *ω*_*e*_versus time *t* during impact for *V*_*i*_ = 61 m s^−1^. As *V*_*i*_ increased from 28 m s^−1^, the peak value of *ω*_*e*_increased to 15,400 rpm, and this decreased significantly to 9,800 rpm just before rebounding. The value of ω^*^(=*ω*_*r*_* *+* ω*_*a*_) is also shown.
